# Pyonephrosis among Patients with Pyelonephritis Admitted in Department of Nephrology and Urology of a Tertiary Care Centre: A Descriptive Cross-sectional Study

**DOI:** 10.31729/jnma.8015

**Published:** 2023-02-28

**Authors:** Lanka Praveen Kumar, Irshad Khan, Amit Kishore, Manoj Gopal, Vineet Behera

**Affiliations:** 1 Command Hospital Central Command, Lucknow, Uttar Pradesh, India; 2 Department of Nephrology, INHS Asvini, Mumbai, Maharashtra, India

**Keywords:** *pyelonephritis*, *pyonephrosis*, *kidneys*

## Abstract

**Introduction:**

Pyonephrosis is a severe complication of pyelonephritis leading to rapid progression to sepsis and loss of renal function resulting in nephrectomy. Early identification of pyonephrosis based on clinical or radiological characteristics amongst pyelonephritis is paramount. This study aimed to determine the prevalence of pyonephrosis among patients with pyelonephritis admitted to the Department of Nephrology and Urology of a tertiary care centre.

**Methods:**

This descriptive cross-sectional study was done in a tertiary care centre among patients with pyelonephritis from 1 July 2016 to 31 Jan 2021. Ethical approval was obtained from Institution Ethics Committee (Reference number: IEC/56/21). The available clinical, demographic and laboratory parameters were recorded from the hospital records in a predesigned proforma. A convenience sampling method was used. Point estimate and 95% Confidence Interval were calculated.

**Results:**

Among 550 pyelonephritis patients, the prevalence of pyonephrosis was 60 (10.9%) (8.313.5, 95% Confidence Interval). The mean age was 54.62±12.14 years, and 41 (68.33%) were males. The most common clinical symptom was flank pain with or without fever in 46 (76.66%) patients. Escherichia coli was the most common offending organism in 20 (33.33%). Ultrasonography showed classical echogenic debris with floaters and internal echoes in 44 (73.33%) patients. Double J stenting was successfully done in 44 (73.33%) patients. Percutaneous nephrostomy was done in the remaining 16 (26.66%) patients.

**Conclusions:**

The prevalence of pyonephrosis in pyelonephritis is similar to previous studies done in similar settings.

## INTRODUCTION

Pyonephrosis is a serious infective condition of kidneys characterised by the presence of pus in the renal collecting system.^1^ It is associated with obstruction in the renal collecting system and suppurative destruction of the renal parenchyma leading to total or near total loss of function of the affected kidney.^2^

Therefore, early diagnosis and prompt management among patients of pyelonephritis is the key to good outcomes.^3^ If the obstruction is relieved early by urinary diversion techniques such as Double J (DJ) stenting or percutaneous nephrostomy (PCN) insertion, and the patient is aggressively managed with antibiotics, there is a possibility of avoiding permanent loss of renal function and subsequent nephrectomy. The existing data is generally in form of case reports or small series with very few existing studies.^4^

This study aimed to determine the prevalence of pyonephrosis among patients with pyelonephritis admitted to the Department of Nephrology and Urology of a tertiary care centre.

## METHODS

This descriptive cross-sectional study was conducted from 1 February 2016 to 31 July 2021 in the departments of Nephrology and Urology in Indian Naval Hospital Ship (INHS) Asvini, Mumbai, India. Data was collected after ethical approval from the Institution Ethics Committee of the same institute (Reference number: IEC/56/21). All adult patients of pyelonephritis aged greater than 18 years visiting the hospital during the study period were enrolled in the study. Patients with infections of the transplanted kidney, pregnant females, or those who did not drain pus from the collecting system after decompression were excluded from the study. Convenience sampling was used. The sample size was calculated by using the following formula:


$$\begin{array}{ll} \text{n} & ={\text{Z}}^{2}\times \frac{\text{p}\times \text{q}}{{\text{e}}^{\text{2}}}\\ & =1.96^{2}\times \frac{0.50\times 0.50}{0.05^{2}}\\ & =385 \end{array}$$

Where,

n = minimum required sample size
Z = 1.96 at a 95% Confidence Interval (CI)
p = prevalence taken as 50% for maximum sample size calculation
q = 1-p
e = margin of error, 5%

The minimum sample size calculated was 385. However, we have included 550 patients in the study.

Pyelonephritis was defined based on Centre of Disease Control (CDC) criteria,^5^ as the presence of clinical features like fever, dysuria, urgency, frequency, costovertebral tenderness with pyuria, or organisms cultured from blood/urine, or evidence of infection on ultrasonography (USG) or Computed Tomography (CT) scan.^6^ Pyonephrosis was defined as evidence of pyelonephritis with radiological presence (by USG or CT scan) of obstruction as hydronephrosis or hydroureteronephrosis (HDUN); or purulent exudate, pus, echogenic debris, fluid/fluid levels in the renal pelvis or urinary collecting system.^7^ After a diagnosis of pyonephrosis, all patients were taken up for urinary diversion with DJ stenting or PCN. The available clinical, demographic and laboratory parameters were recorded as per the proforma. The standard cut-offs for the laboratory of this centre were considered for any abnormalities. Anaemia was defined as haemoglobin less than 12 g/dL, leukocytosis as leucocyte count ≥11500/cubic mm, azotemia as serum creatinine ≥1.4 mg/dL and pyuria as urine leucocytes ≥10/HPF.

Data were analysed using IBM SPSS Statistics 21.0. Point estimate and 95% CI were calculated.

## RESULTS

Among 550 patients, pyonephrosis was found in 60 (10.90%) (8.30-13.50, 95% CI). The mean age was 54.62±12.14 years, and the majority of them were males 41 (68.33%). The most common clinical symptom was flank pain with or without fever, seen in 46 (76.66%) patients. Fever was present in 37 (61.66%) patients The most common haematological abnormalities were leucocytosis seen in 49 (81.66%) and anaemia seen in 46 (76.66%) cases. The urine was cloudy in 48 (80%) patients and frank pus was drained in 12 (20%) patients (Table 1).

**Table 1:** Baseline characteristics and clinical presentation of patients with pyonephrosis (n= 60).

Characteristics	n (%)
**Sex**	
Male	41 (68.33)
Female	19 (31.66)
**Age group (years)**	
<50	19 (31.66)
>50	41 (68.33)
**Underlying medical comorbidities**	
Hypertension	6 (10)
Diabetes	18 (30)
Diabetes and hypertension	5 (8.33)
Chronic kidney disease	17 (28.33)
Overweight	38 (63.33)
HIV^*^ positive	1 (1.66)
**Affected side**	
Right kidney	25 (41.7)
Left kidney	35 (58.3)
**Clinical presentation**	
Fever with flank pain	24 (40)
Fever with dysuria	6 (10)
Fever with pyuria	7 (11.66)
Flank pain and dysuria	5 (08.33)
Flank pain alone	17 (28.33)
Sepsis with shock	1 (1.66)
**Laboratory abnormalities**	
Anemia	46 (76.66)
Leucocytosis	49 (81.66)
Pyuria	57 (95)
Azotemia	21 (35)

* HIV: human immune deficiency virus; ^+^HPF: high power field

*E. coli* was the most common offending organism seen in 20 (33.33%) followed by *Pseudomonas* in 9 (15%), while no growth was seen in 10 (16.66%) patients (Table 2).

**Table 2:** Distribution of microorganisms in urine culture from the first urine sample (n= 60).

Organism isolated	n (%)
E. coli	20 (33.33)
Pseudomonas species	9 (15)
Klebsiella pneumonia	5 (8.33)
Proteus species	4 (6.66)
Staphylococcus aureus	3 (5)
Acinetobacter baumanii	2 (3.33)
Mixed growth (≥2 organisms)	7 (11.66)
No growth	10 (16.66)

X-ray KUB showed radiopaque calculi in the region of the ureter in 19 (31.66%), of which 12 (63.15%) were in the proximal and 7 (36.84%) were in the distal ureteric region. A total of 16 (26.66%) patients had radiopaque calculi in the renal fossa, and 8 (50%) were staghorn calculi (occupying the entire pelvis or at least two calyces). Ultrasonography showed classical echogenic debris with floaters and internal echoes in the dilated pelvicalyceal system in 44 (73.33%) patients. A total of 29 (48.33%) patients underwent non-contrast CT scans and 31 (51.66%) underwent contrast-enhanced CT (ce-CT) scans with the urography phase. CT scan revealed features of ureteric obstruction in 19 (31.66%) patients.

DJ stenting was successfully done in 44 (73.33%) patients. PCN was done in the remaining 16 (26.66%) patients. Nephrectomy was done in 6 (10%) cases. A total of 1 (1.66%) patient who presented with features of sepsis and septic shock, was treated aggressively with antibiotics, supportive measures, and PCN to drain about 650 ml of pus; but he succumbed to his illness.

## DISCUSSION

The present study showed that pyonephrosis was found in 10.90% of pyelonephritis. E. coli was the most common offending organism in this study. The classic ultrasonography features included echogenic debris with floaters and internal echoes in the dilated pelvicalyceal system in 44 (73.33%) patients. A previous study showed 17 (10%) patients had pyonephrosis amongst pyelonephritis, most probably secondary to a stone. These findings are similar to the findings of our study.^8^ Pyonephrosis represents a spectrum of infected diseases of the kidney ranging from infected hydronephrosis to the more diffuse xanthogranulomatous pyelonephritis.^9^ It is characterised by a collection of purulent material in the pelvicalyceal system,^10^ due to any form of distal obstruction.^11^ The most common aetiology is a stone in the ureter or kidney.^12^

A previous study extensively studied the role of USG in pyonephrosis kidneys showing a spectrum of USG findings ranging from echogenic debris to solid-looking material in the pelvicalyceal system seen in 61% of cases.^13^ In our study, USG could diagnose pyonephrosis in 73.3% of patients. Another study found radiological features of pyonephrosis in 12% of pyelonephritis patients.^13^ CT scan is the radiological modality of choice and the presence of gas or fluid/ fluid levels in the pelvicalyceal system is strongly suggestive of an infective aetiology in CT, with other findings being the thickening of the renal pelvis with perinephric fat stranding.^7,14^

Gram-negative organisms especially *E. coli* are the most commonly isolated organism in patients with pyonephrosis with the incidence of E Coli being 28.5 % (28/70) in a study.^15^ Our study showed a similar cultural profile. The initial management in pyonephrosis is urgent decompression of the urinary system, by either PCN or ureteral stenting with DJS, with neither of them showing superiority in terms of the effectiveness of drainage.^2,3^

As the data were collected retrospectively, there might be missing data. There is also a likelihood of measurement bias amongst clinicians and radiologists in diagnosis cases of pyonephrosis. Since the convenience sampling method was used so there might be selection bias and could not be generalized in a larger population.

## CONCLUSIONS

The prevalence of pyonephrosis was similar to other studies done in similar settings. The patients with pyonephrosis have flank pain and fever as the common clinical features and *E. coli* is the commonest offending organism. Early detection and management of pyonephrosis is a most in patients with pyelonephritis.
